# Chemistry of the Metals—Silver and Its Compounds

**Published:** 1853-10

**Authors:** R. N. Wright


### AMERICAN JOURNAL

OF

### DENTAL SCIENCE.

Vol. IV. NEW SERIES—OCTOBER, 1853. NO. 1.

### ARTICLE I.

*Chemistry of the Metals—Silver and its Compounds.* By Professor R. N. Wright, M. D.

(Continued from page 565.)

*Salts of Silver.*—We propose as the conclusion of the article which appeared in the last number of the Journal, to describe the remaining compounds and combinations of silver, and the method of electro-plating as practiced by Mr. Smee, together with the process of assaying.

The first combinations to be described are those formed between silver in the shape of oxyd, and acids; and, the first we will examine is that compound formed by a union of carbonic acid and oxyd of silver known as

*Carbonate of Silver.*—The readiest method for the formation of this substance is, to add to a solution of *nitrate of silver* in distilled water, a small quantity of carbonate of potash also in solution, there is a rapid interchange of elements—the nitric acid of the silver, taking up with the potash and the carbonic acid of the potash uniting with the oxyd of silver. The new substance, when first formed in this manner, appears as a white flocculent precipitate, but speedily blackens on exposure to light.

*Sulphate of Silver.*—This salt is formed abundantly, whenever we mix a soluble alkaline sulphate (as (e. g.) sulphate of soda) in solution, with a solution of nitrate of silver, or it may be made by pouring sulphuric acid upon silver, and then applying heat. The precipitate has a white color and saline taste, and is capable of crystallization—the crystals being prismatic and containing little or no water, indeed, when properly prepared they are quite auhydrous, requiring about 91 times their weight of water, at 60° F. for perfect solution. Water at 212° F. possesses a much greater solvent power; there is, however, little advantage in using it, since all the excess is deposited when the solution again reaches 60° F.

It is asserted that "upon a large scale, small portions of gold may be most economically separated from large quantities of silver, by heating the finely granulated alloy in sulphuric acid; the gold remains in the form of a black powder, and the sulphate of silver may be decomposed by the action of metallic copper; the silver is precipitated in a pulverulent state, and, with a little borax or other vitrifiable flux, is fuzed, and cut into ingots; the sulphate of copper is easily obtained in the crystallized state, by evaporating the residuary liquid." "A compound acid, which may be called *nitro-sulphuric,* consisting of 1 part of nitre dissolved in about 10 of sulphuric acid, dissolves silver at a temperature below 200°, and the solution admits of moderate dilution before sulphate of silver separates from it. This acid, scarcely acts upon *copper*, *lead* or *iron*, unless diluted with water; it is, therefore, useful in separating the silver from old plated articles; the precious metal, may afterward be separated, either in the form of chloride, by adding common salt, or by diluting the acid, and continuing the immersion of the pieces of copper which have lost their silvering, and which will now dissolve in the diluted acid, and occasion the precipitation of metallic silver."

Sulphate of silver, will be decomposed by a heat a little below absolute redness, and metallic silver remains. The best form of crystallization is obtained by adding diluted sulphuric acid to its solution.

*Hyposulphate of Silver.*—The best process for the formation of this salt is that recommended by Heeren; viz. to digest carbonate of silver, (formed by the process we have described, or any other more convenient,) in hyposulphuric acid, a permanent crystalline mass is the result, which is quite soluble, requiring only about two parts of water for its solution. The *ammonio-hyposulphate* of silver will be formed, when ammonia in considerable quantity is added to the solution, the result being a mass of minute, granular crystals.

*Sulphite of Silver.*—By digesting oxyd of silver in sulphurous acid, we produce a granular mass, which is sulphite of silver; and it may also be obtained through the agency of double decomposition, by adding a soluble sulphite of an alkali, to a soluble salt of silver, as the nitrate.

*Hyposulphite of Silver.*—The following description of this salt may be found in Brande, p. 962. He says, "it has been examined by Hershell, and is formed by dropping a weak solution of nitrate of silver into a very dilute solution of *hyposulphite of soda:* a white cloud is at first produced, which redissolves on agitation; on adding more of the precipitant, the cloud reappears, and aggregates into a gray precipitate, which appears to consist of *hyposulphite of silver;* the supernatant liquor tastes intensely sweet, which is remarkable, considering the disgusting bitterness both of the nitrate and of the hyposulphite, and shows 'how little we know of the way which bodies affect the organs of taste. Sweetness and bitterness, like acidity, seem to depend upon no particular principle, but seem to be regulated by the state of combination in which the same principles exist at different times.' *Hyposulphite of silver* is also produced, when chloride of silver is dissolved in any of the hyposulphites, the solution is intensely sweet without any metallic flavor. These facts show the strong affinity that exists between *oxyd of silver* and *hyposulphurous acid.* The solubility of argentine compounds, in hyposulphite, has led to an important application of them in photogenic drawing, for the purpose of fixing the designs, by the removal of all adhering or unchanged salt of silver. *Hyposulphite of silver* is very prone to decomposition, so as to form sulphate of oxyd of silver, and sulphuret of silver; hence the occasional blackening of its solutions."

*Nitrate of Silver.*—When silver is exposed to the action of *nitric acid*, diluted with about three parts of water, there is a rapid disengagement of *nitric oxyd gas*, and a corresponding diminution in the bulk of the silver, because the acid is dissolving it, and the characteristics of the result will depend much upon the purity of the acid used in the experiment; there will be a cloudy solution and white precipitate; if *muriatic acid* be present, the presence of gold will give a dark colored powder of undissolved metal, and if copper is present, we shall have a solution of a bluish-green color.

When we have obtained a solution of nitrate of silver, free from the above and other impurities, it is perfectly transparent and exceeding caustic, (if the solution be a saturated one.) From a property which it possesses, of changing all organic textures with which it comes in contact, to a purplish black color, which is permanent, it is extensively used as the basis of indelible inks. As we procure it in the shops, in small circular sticks, it is far from being pure, and only semi-crystalline; but it may be beautifully crystallized into plates, and when thus dissolved in distilled water, its properties can be well displayed. This salt is very soluble in water, demanding only about an equal weight at 60° F., and whenever the solution is required for accurate experiments, the water should be carefully distilled. When well prepared, the salt exhibits no acid properties with the ordinary tests, but is strictly neutral, and the solution should be kept in well stopped vials, of a dark color, and in a place to which light cannot gain access, otherwise, by exposure, it becomes speedily blackened.

Brande says, "when nitrate of silver has been thus acted upon by light, it is no longer perfectly soluble in water, owing to the separation of a portion of metallic silver." The best method of prepairing the common "lunar caustic" of the shops, is to expose the nitrate to the action of heat in silver crucibles, it then becomes a crystalline mass, having a grayish-white color, and is commonly sold in cylindrical sticks. There is some care necessary in its production, such as keeping the moulds well warmed, but not too hot, a high temperature being apt to blacken it, and if the temperature be carried to redness, the salt will be decomposed with the reduction of metallic silver.

"Sulphur, phosphorus, charcoal, hydrogen and several of the metals decompose this nitrate. A few grains mixed with a little sulphur, and struck with a heavy hammer upon an anvil, will give a detonation; phosphorus occasions violent explosion when about half a grain of it is placed upon a crystal of the nitrate, upon an anvil, and struck sharply with a hammer; and if heated with charcoal it deflagrates, and the metal is reduced."

The immersion of many substances into a solution of nitrate of silver, will occasion decomposition, with the revival and deposition of the metal. A clean stick of phosphorus, thus immersed, will speedily become covered with a beautiful arborescent incrustation. Deposition also rapidly takes place upon slips of clean metal, as copper, &c.

"*Mercury introduced into the solution of nitrate of silver, causes a beautiful crystalline deposit of silver, called the *arbor Dianæ;* as remarked by Lemery. To obtain this crystallization in its most perfect state, the solution should contain a little mercury, and the mercury put into it should contain a little silver. Baumé directs an amalgam of one part of silver with seven of mercury, of which, a small piece is to be introduced into a solution composed of 6 drachms of saturated nitrate of silver, and 4 drachms of a similar solution of mercury, diluted with 5 ounces of distilled water: a small flask or matrass should be used for the experiment, kept perfectly at rest; in a few minutes small filaments of silver darken the surface of the amalgam, and in about eight and forty hours, the whole has separated in a shrub-like form. The addition of mercury to the solution, and of silver to the precipitating mercury, is said to give a degree of tenacity to the arborescent deposit of crystals, which prevents their falling to the bottom of the flask."

*Brande, p. 960.

Ammonia added to a solution of nitrate of silver occasions a yellowish-white precipitate, which is soluble in excess of the precipitant; and we thus produce a valuable auxiliary fluid-test for the presence of *arsenic*, known as the *ammonio-nitrate of silver* test. In preparing it, the ammonia, should be added, drop by drop, until the precipitate is on the eve of being dissolved, when it is ready for use.

Nitrate of silver is decomposed with precipitation by many of the strong acids, by many of the metallic salts, and also by most of the alkaline oxyds. We have already remarked, that this salt forms the basis of most of the indelible inks; the following is, perhaps, as good a recipe as can be used: "Dissolve* 2 drachms of nitrate of silver, and 1 drachm of gum arabic, in 7 drachms of distilled water, colored with a little China ink. The preparatory liquid for moistening the cloth, is made by dissolving 2 ounces of crystallized carbonate of soda and 2 drachms of gum arabic in 4 ounces of water. Nitrate of silver is an ingredient in some of the hair dies, is an escharotic in surgery, and is administered internally in medicine, with this bad effect, however, that if exhibited for any length of time, it imparts, by absorption into, and discoloration of, the *rete mucosum*, an olive brown tint to the whole surface of the body, which is permanent and unchangeable."

Grahame says, that on account of the expense of the preparation for writing on linen, which we have described, bleachers have substituted a solution of coal tar in naptha, made sufficiently thin for the pen, and which will effectually resist the action of chlorine.

As a test for the presence of chlorine, whether free or combined, nitrate of silver is one of the best we possess; producing immediately a dense flocculent precipitate, which subsides, white at first, but speedily changing color; passing through various shades of purple to a dark color; becoming almost black on exposure to light.

There are other substances which will produce similar pre-

*Brande, p. 960.

cipitates with nitrate of silver, but we will generally have little trouble in ascertaining their nature. Hydrobromic, hydriodic and hydrocyanic acids are mentioned, as belonging to the list of those substances which produce white precipitates with nitrate of silver, blackening on exposure to light.

*Nitrite of Silver.*—The following is Proust's method of obtaining this substance: Saturate good nitric acid with silver, and then boil in it silver reduced to fine powder; or *nitrate of soda* may be fused until it becomes nitrite, and in this condition, may be added to a clear solution of a silver salt, the combination resulting in the production of a brown precipitate; which is to be dissolved in water at a temperature of 212° F., precipitated by nitrate of silver, and filtered at a high temperature; the cooling of the solution separating nitrite of silver, a very sparingly soluble salt, requiring at 60° F. more than 100 times its weight of water for solution.

*Chlorate of Silver* is produced whenever oxyd of silver is digested in chloric acid, and appears in the shape of rhombic crystals sparingly soluble in water.

*Iodate of Silver* is the result of the admixture of a solution of a soluble salt of silver, and iodic acid or a solution of a soluble alkaline iodate, a white precipitate falling, which is decomposed on the addition of *sulphurous acid*, with the production of *sulphuric acid* and *iodide of silver.*

*Cyanate of Silver.*—According to Liebig, when cyanate of potash is dropped into a solution of nitrate of silver, a white precipitate, soluble in water at 212° F. is thrown down, which will burn with deflagration, blacken with a high temperature, and result in *cyanic acid, carbonic acid, nitrogen* and *dicyanuret of silver;* it is supposed to be a true cyanate of silver.

*Borate of Silver* is formed readily by adding a solution of boracic acid to a solution of nitrate of silver, and the result is but sparingly soluble in water.

*Phosphate of Silver* is produced whenever we add a solution of some alkaline phosphate, (as phosphate of soda,) to a solution of nitrate of silver. It has a yellowish color, and blackens on exposure to light.

*Pyrophosphate of Silver* is formed by a process analogous to the last described, only substituting *pyrophosphate of soda* for the phosphate; its color, however, is different, being white instead of yellow.

*Chromate of Silver* is produced whenever we add a solution of an alkaline chromate, as chromate potash or soda, to a solution of nitrate of silver; its color being at first deep red, changing to a dark brown on exposure to light.

*Arsenite of Silver* is the result of a union of *arsenious acid* and nitrate of silver, both in solution, and the *arseniate of silver* can be formed in the same manner by substituting *arsenic acid* for *arsenious.*

*Fulminate of Silver.*—For the subjoined description of this substance, see Brande, page 965. "This curious and dangerous compound, is prepared as follows: 100 grains of fused and finely powdered nitrate of silver, are added to an ounce of warm alcohol, and the mixture stirred in a sufficiently large glass basin; an ounce of *fuming nitric acid* is then added, and presently a violent effervescence ensues, and a powder falls; as soon as this appears white, cold water is added, and the powder is immediately to be collected upon a filter, washed and carefully dried at a temperature of 100° F. In collecting and handling this powder, the utmost caution is requisite; it should be made in small quantities only, and touched with nothing hard, for it has sometimes exploded upon the contact of a glass rod, even under water: the feather of a common quill serves to collect it; and it should be kept in a wide-mouthed vessel, covered by paper, and by no means in a stoppered, or even in a corked, vial, as serious accidents have arisen from its unexpected explosion. In short, one cannot be too careful in meddling with it, and its use for fulminating balls, and other purposes of amusement is highly dangerous. Berzelius observes, that, in preparing fulminating silver, a vessel of sufficient capacity should be used to prevent the liquid running over during effervescence, by which portions of the powder are deposited upon its exterior, and apt to explode when dry; that all approach of flame should be avoided, during the escape of *nitrous etherized gas*, because its inflammation would probably occasion the powder to explode; and that care should be taken, to avoid introducing all hard substances, to stir or touch the precipitate."

He describes Liebig's process as follows: "A drachm of refined silver, is dissolved in half an ounce of nitric acid, specific gravity, 1.36 to 1.38; two ounces of alcohol, specific gravity, 0.85, are then added, and the whole heated in a matrass; white flocculi soon appears, and when ebullition begins, the heat is to be withdrawn; the effervescence, however, continues, and the powder falls; when action ceases, the powder is to be collected with the precautions above described."

Fulminating silver is mentioned as "a gray crystalline powder; acquiring a dingy hue by exposure to light, dissolving in about 40 times its weight of water at 212° F., and depositing minute crystals, as the solution cools. From one-half a grain to a grain will detonate violently, either by means of heat, friction, percussion, sulphuric acid, galvanism or electricity."

We have now collected and arranged a brief summary of all the important compounds of silver, rather with the view of making the article systematically complete, than with any other object; and, we will now proceed to a conclusion, with the description of the process of *electro-plating*, the method of assaying silver, and, the characteristics of its salts.

*Galvano-Plating.*—"The method of *silvering* or *plating* by the galvanic current," says Mr. Smee, "is difficult, but the general plan of action very nearly resembles that pointed out in the article on *galvano-gilding*. The surface of the object we desire to coat, must be cleaned by a solution of potassa, and then rubbed over with whiting; after which, it is ready for insertion in the fluid. The galvanic arrangements are similar to those for gilding, with the exception, that instead of a platinum wire, a silver wire should be used as the oxygen pole or anode, or in other words, that in connection with the silver of the battery. The object to be plated, (as in the case of gold, already described,) must not remain a single instant in the solution, whilst the circuit is incomplete. All further arrangements are similar to those described for gold.

The solution used must either be a weak *sulphate*, *acetate* or

*hyposulphite* of silver, with a little *dilute sulphuric acid.* After the metal has been in the solution for two or three minutes, it will assume a dark color, when it is to be removed, and rubbed over with whiting, and this process repeated till a sufficient thickness of metal is obtained. The regulation of the quantity of *electricity* is important, and is best effected by using the finest possible piece of silver wire as the positive pole."

"In the description given for coating other metals with a thin layer or film of any of the noble metals, a weak solution has been recommended, as less likely to favor local action; but if we are desirous of coating any metal, where no elective affinity can take place, the metallic solution may be used of any strength."

*Assay of Silver.*—The following description of this process is copied, verbatim, from Brande, page 970. "The analysis of alloyed silver, is a very important process, and in continual practice by refiners and assayers. It may be performed in the humid way by dissolving the alloy in *nitric acid*, precipitating with *hydrochloric acid*, or *chloride of sodium*, and either reducing the chloride by potassa, or estimating the quantity of silver it contains. The usual method, however, which is employed at the mint, and by the refiners, is cupellation. Of the useful metals, there are three capable of resisting the action of the air, at high temperatures; these are silver, gold and platinum; the others, under the same circumstances, become oxydized; it might, therefore, be supposed, that an alloy containing one or more of the first three metals, would suffer decomposition, by mere exposure to heat and air, and that the oxydizable metal, would burn into oyxd. This, however, is not the case; for if the proportion of the latter be small, it is protected, as it were, by the former; or, in other cases, a film of infusible oxyd, coats the fused globule, and prevents the further action of the air. These difficulties are overcome by adding to the alloy some highly oxydizable metal, the oxyd of which is fusible. Lead is the metal usually selected for this purpose, though bismuth, will also answer. Supposing, therefore, that an alloy of silver and copper is to be assayed, or analyzed by cupellation; the following is the mode of proceeding: a clean piece of the metal, weighing about 20 grains, is laminated and accurately weighed in a very sensible balance. It is then wrapped up in the requisite quantity of sheet lead, (perfectly pure and reduced from *litharge;*) apportioned by weight to the quality of the alloy under examination, and placed upon a small cupel or shallow crucible, made of bone earth, which has been previously heated. The whole is then placed within the muffle, heated to bright redness: the metals melt, and, by the action of the air which plays over the hot surface, the lead and copper are oxydized and absorbed by the cupel, and, if the operation has been skillfully conducted, a button of pure silver ultimately remains; the completion of the process being judged of by the cessation of the oxydation, and motion upon the surface of the globule, and by the very brilliant appearance assumed by the silver when the oxydation of the alloy ceases. The button of pure metal is then suffered to cool gradually; and its loss of weight will be equivalent to the weight of the alloy, which has been separated by oxydation, a certain allowance being made for a small loss of silver, which always occurs, partly by evaporation and partly carried off by the oxyds which are absorbed by the cupel. To perform this process with accuracy, certain precautions are requisite, which can only be learned by practice, so as to enable the operator to gain uniform results."

*Characters of the Salts of Silver.*—The author we have just quoted, in describing the characters of the salts of silver, says, "the soluble of salts of silver are recognized by furnishing a white precipitate with hydrochloric acid, and the soluble chlorides, which blackens by exposure to light, and which is readily soluble in ammonia; and by affording metallic silver, on the immersion of a plate of copper. The salts, insoluble in water, are mostly soluble in liquid ammonia; when heated on charcoal, before the blowpipe, they afford a globule of silver. All the salts of silver, excepting those which contain colored acids, are colorless, provided, they have not been exposed to light, or deoxydizing agents, of the influence of which, they are extremely susceptible. A *yellow precipitate* on the addition of phosphate of soda, and of the soluble *arsenites*, a *red brown* by *arseniates*, a *crimson* by chromates, and *white* by *fenocyanuret of potassium*, are further characteristics of the soluble salts of silver." "*Tin* and *lead*, are the most rapid *precipitants* of metallic silver from the nitrate; *cadmium*, *zinc*, *copper*, *bismuth* and *antimony*, are more slow in their operation, and *arsenic* and *mercury* still more tardy. In all cases, the silver appears crystallized; often, blackish at first, but afterwards assuming the metallic lustre. *Iron* is a speedy reducer of the *sulphate of silver*. The insoluble salts of silver mixed with water, are also similarly decomposed, but the operation is more slow. *Chromate of silver*, probably, on account of its insolubility, is extremely slowly reduced; *cadmium* is the most effectual metal for the purpose. *Chloride of silver* is rapidly reduced, by most of the metals which form *soluble chlorides*, such as *zinc*, *iron*, *cadmium*, *cobalt* and *arsenic; lead*, *nickle*, *copper*, *antimony* and *mercury*, act slowly; and *tin* and *bismuth*, are very feeble in their action. *Zinc*, *copper* and *arsenic*, rapidly deduce the ammoniacal solution of oxyd of silver. Of all the *metallic* precipitants, *zinc* and *cadmium* are the most effective; but, when *zinc* or *antimony* are used, the separated *silver* contains those metals."

The subject of the next article will be platinum.

[To be continued.]

### ARTICLE II.

*Patents and Patentees.* By J. W. Keyes, M. D., D. D. S., of Quincy, Fla.

